# Shared psychological characteristics that are linked to aggression between patients with Internet addiction and those with alcohol dependence

**DOI:** 10.1186/1744-859X-13-6

**Published:** 2014-02-21

**Authors:** Jae Yeon Hwang, Jung-Seok Choi, Ah Reum Gwak, Dawn Jung, Sam-Wook Choi, Jaewon Lee, Jun-Young Lee, Hee Yeon Jung, Dai Jin Kim

**Affiliations:** 1 Department of Psychiatry, Kangdong Sacred Heart Hospital, Hallym University College of Medicine, Seoul 134-814, Republic of Korea; 2 Department of Psychiatry, SMG-SNU Boramae Medical Center, Seoul 156-707, Republic of Korea; 3 Department of Psychiatry, Seoul National University College of Medicine, Seoul 110-799, Republic of Korea; 4 Department of Psychiatry, Gangnam Eulji Hospital, Eulji University, Seoul 135-010, Republic of Korea; 5 Department of Psychiatry, Seoul St. Mary's Hospital, The Catholic University of Korea College of Medicine, Seoul 137-701, Republic of Korea

**Keywords:** Internet addiction, Alcohol dependence, Aggression, Personality, Impulsiveness, Anger

## Abstract

**Background:**

Internet addiction (IA) is considered as one of behavioral addictions. Although common neurobiological mechanisms have been suggested to underlie behavioral addiction and substance dependence, few studies have directly compared IA with substance dependence, such as alcohol dependence (AD).

**Methods:**

We compared patients with IA, AD, and healthy controls (HC) in terms of the Five Factor Model of personality and with regard to impulsiveness, anger expression, and mood to explore psychological factors that are linked to aggression. All patients were treatment-seeking and had moderate-to-severe symptoms.

**Results:**

The IA and AD groups showed a lower level of agreeableness and higher levels of neuroticism, impulsivity, and anger expression compared with the HC group, which are characteristics related to aggression. The addiction groups showed lower levels of extraversion, openness to experience, and conscientiousness and were more depressive and anxious than the HCs, and the severity of IA and AD symptoms was positively correlated with these types of psychopathology.

**Conclusions:**

IA and AD are similar in terms of personality, temperament, and emotion, and they share common characteristics that may lead to aggression. Our findings suggest that strategies to reduce aggression in patients with IA are necessary and that IA and AD are closely related and should be dealt with as having a close nosological relationship.

## Background

Internet addiction (IA) is considered a behavioral addiction [1], and behavioral addictions, such as pathological gambling, share core features with substance addiction, such as alcohol dependence (AD). These shared features include continued engagement in addictive behavior despite adverse consequences, diminished self-control over engagement in such behavior, and an appetitive urge or craving state prior to engagement in the behavior [2]. However, few studies have investigated the similarities between IA and substance addiction, which would be necessary to incorporate IA into addiction disorder.

The relationship between AD and aggression is well known. Acute alcohol use is related to about a half of all violent crimes and sexual assaults, and it increases the risk of domestic partner violence [3]. Similarly, the relationship between IA and aggression has been investigated. Previous studies suggest that being male, being depressed, and having low self-esteem and poor family functioning are associated with IA [4-6] and aggression [7-9]. Furthermore, an association between IA and aggressive behavior among adolescents has been reported [10]. The Internet offers anonymous activities, such as chatting, online gaming, and gambling, in which personal responsibility is decreased. Online gaming creates high arousal, sensory overload, and novel situations. These factors, which are inherent in online gaming, or harmful Internet use in general, may contribute to aggressive behavior online [10,11]. Understanding how IA is linked to aggression and violent behavior is crucial for the development of strategies to prevent violence.

Although it is evident that IA and AD are associated with aggression, aggression itself is moderated by a complex psychological process. Anger is one of the emotional components of aggressive behavior when it is expressed in a destructive way [12]. Impulsivity is a long-established underlying psychological construct related to aggressive behavior, addictive behavior, and suicide [13]. Neuroticism with regard to the Big Five Model of personality is positively correlated with reactive aggression, which may be caused by anger, whereas the personality trait of extraversion is correlated with proactive aggression, which may be used to achieve goals. Agreeableness is negatively correlated with both types of aggression [14].

Therefore, we compared patients with IA with those with AD in terms of psychological factors that could lead to aggressive behaviors, such as anger, impulsivity, and personality characteristics from the Five Factor Model. We hypothesized that if IA and AD shared common characteristics linked to aggression, patients in both groups would show high levels of anger expression, impulsivity, and neuroticism and a low level of agreeableness compared with healthy control subjects. Linking IA with AD by showing shared characteristics may help to clarify the nosology of IA which has been controversial since its development.

## Methods

We enrolled 30 patients diagnosed with IA (mean age, 22.67 ± 6.69 years), 30 patients diagnosed with AD (mean age, 30.03 ± 5.89 years), and 30 healthy controls (HCs, mean age, 25.33 ± 2.77 years). All patients were treatment-seeking, visiting our clinic complaining of compulsive Internet use, or having alcohol-related problems. We restricted enrollment to male patients and healthy controls because males have a higher prevalence of problematic Internet use than females do [15,16]. Patients were recruited from the outpatient clinic of the Seoul Metropolitan Government-Seoul National University (SMG-SNU) Boramae Medical Center in Seoul, South Korea.

Patients with IA were assessed using the standardized Korean version of Young's Internet Addition Test (IAT) [17,18]. We recruited patients with IAT scores of at least 70 who spent more than 4 h per day and 30 h per week using the Internet. Although previous studies have defined excessive Internet use as an IAT score of at least 50 [19,20], our inclusion criterion was stricter to ensure our sample had severe IA, rather than being at high risk for IA. Young's IAT is in accordance with the DSM-IV criteria for pathological gambling and is widely used by investigators across the world. Test items are rated on a 5-point scale in which 1 indicates ‘very rarely’ and 5 indicates ‘very frequently’. Total scores were calculated according to Young's method, and possible scores for all 20 items ranged from 20 to 100 [17].

AD was diagnosed using the Structured Clinical Interview for DSM-IV (SCID) [21]. The Alcohol Use Disorder Identification Test-Korean version (AUDIT-K) [22] was used to assess the severity of alcohol use disorders.

The SCID was used to identify past and current psychiatric illnesses. Of the 30 patients diagnosed with IA, 5 fulfilled the DSM-IV criteria for depressive disorders and 2 fulfilled them for anxiety disorders. All patients with IA used the Internet primarily for online gaming. HCs were recruited from the local community and had no history of psychiatric disorders. Subjects were enrolled in the AD and HC groups if they used the Internet less than 2 h per day. All patients were drug-naïve. The Beck Depression Inventory (BDI) [23] and Beck Anxiety Inventory (BAI) [24] were used to assess depressive and anxious symptoms, respectively, in all participants.

Personality was assessed using the short form of the Korean version of the NEO Personality Inventory-Revised (NEO-PI-R) [25,26]. The NEO-PI-R [27] assesses personality characteristics according to the Big Five Model of personality and is widely used in research related to personality. The Big Five Model evaluates five dimensions of personality: neuroticism (includes traits such as being tense, moody, and anxious), extraversion (includes specific traits such as being talkative, assertive, and energetic), agreeableness (includes traits such as being sympathetic, affectionate, and kind), openness to experience (includes traits such as having wide-ranging interests, and being imaginative and insightful), and conscientiousness (includes traits such as being organized, thorough, and competent) [15]. A summarized explanation of these five personality constructs were described elsewhere [28].

Impulsivity was measured using the Korean version of the Barratt Impulsiveness Scale-Version 11 (BIS-11) [29,30]. The BIS-11 [31,32] consists of three factors: cognitive impulsiveness (making quick decisions), motor impulsiveness (acting without thinking), and nonplanning impulsiveness (a lack of ‘futuring’ or foresight). Researchers have accepted the validity of this measure [33].

Anger was assessed using the Korean version of the State–Trait Anger Expression Inventory (STAXI-K) [34,35]. This inventory measures anger experience and anger expression separately; however, we were interested only in the expressive domain of anger and, thus, included only the 20 items measuring anger expression.

Although the data on all variables, with the exception of those on education and state anger expression collected from HCs, conformed to a Gaussian distribution, we performed a nonparametric Kruskal–Wallis test to compare the distributions of continuous variables across the three groups because the within-group variances were not equal for most variables. The *post hoc* analysis was performed using a nonparametric Mann–Whitney *U* test and a Bonferroni correction for multiple comparisons involving the three groups. Pearson's correlation analysis was used to identify correlations between symptom severity and various factors in the IA and AD groups. All statistical analyses were performed using the Statistical Package for the Social Sciences version 20.0 (SPSS Inc., Chicago, IL, USA). A *p* value of 0.05 was deemed statistically significant. The Institutional Review Boards of the SMG-SNU Boramae Medical Center approved the study protocol, and all subjects provided written informed consent prior to participation.

## Results

The mean duration of illness was 11.17 ± 5.64 years in the IA group and 12.67 ± 6.10 years in the AD group. The mean IAT score for the IA group was 76.33 ± 5.79, and the mean AUDIT-K score for the AD group was 25.07 ± 6.70. The mean number of hours of Internet use per day and per week in the IA group was 6.75 ± 2.86 and 47.61 ± 15.83, respectively. The mean amount of alcohol consumed per day by the AD group was 12.60 ± 4.65 drinks. These characteristics indicate that patients in both groups had moderate-to-severe problems.

Although education years did not differ across groups (*p* = 0.131), age did significantly differ across groups (*p* < 0.001). The *post hoc* test revealed that patients with AD were the oldest and patients with IA were the youngest.

Patients with IA or AD were significantly more depressed and anxious than were HCs (*p* < 0.001 for the BDI and BAI). Thus, the IA and AD groups were similar in this regard (Table 1).

**Table 1:** Comparison of clinical characteristics, personality, and temperament between patients with Internet addiction, those with alcohol dependence and healthy controls

	**Internet addiction (*N*** **= 30)**		**Alcohol dependence (*N*** **= 30)**		**Healthy control (*N*** **= 30)**		***p *** **value**	***Post hoc *** **tests**
	**Mean**	**SD**	**Mean**	**SD**	**Mean**	**SD**		
Age (years)	22.67	6.69	30.03	5.89	25.33	2.77	<.001	A > H > I
Education (years)	15.47	8.00	13.40	2.37	14.80	1.67	.131	
Duration of illness	11.17	5.64	12.67	6.10				
BDI	17.00	10.90	22.73	13.79	3.43	4.34	<.001	I = A > H
BAI	16.07	12.73	18.67	13.59	3.77	3.63	<.001	I = A > H
IAT	76.33	5.79						
AUDIT-K			25.07	6.70				
NEO-PI-R								
Agreeableness	38.03	5.78	37.33	6.09	42.63	4.55	.001	H > I = A
Conscientiousness	33.33	8.44	36.43	9.93	42.50	5.09	<.001	H > I = A
Extraversion	34.87	7.71	36.37	8.50	42.27	4.13	<.001	H > I = A
Neuroticism	39.13	7.11	39.07	8.37	28.50	6.69	<.001	I = A > H
Openness to experience	36.70	5.31	36.47	4.08	39.83	4.21	.008	H > I = A
BIS-11								
Cognitive	18.33	3.63	19.50	3.85	16.43	2.42	.006	A > I = H
Motor	22.83	6.90	23.37	7.29	17.47	4.71	<.001	I = A > H
Nonplan	27.07	5.75	28.57	5.35	22.67	2.56	<.001	I = A > H
Total	68.40	14.95	71.43	14.39	56.57	7.59	<.001	I = A > H
Anger expression								
State	26.07	14.15	22.67	15.23	10.77	1.50	<.001	I = A > H
Trait	22.90	5.02	23.70	8.18	17.70	5.48	.001	I = A > H

Kruskal–Wallis test was used to compare distribution of ranks across the three groups. For *post hoc* comparison, Mann–Whitney *U* test with a Bonferroni correction was used.

The three-group comparison and *post hoc* analyses revealed that patients with IA or AD showed a lower level of agreeableness, conscientiousness, extraversion, and openness to experience and a higher level of neuroticism on the short version of the NEO-PI-R than did the HC group (*p* = 0.001, *p* < 0.001, *p* < 0.001, *p* = 0.008, and *p* < 0.001, respectively). Moreover, the NEO-PI-R results did not significantly differ between the IA and AD groups in the *post hoc* analyses.

The total BIS-11 score, a measure of overall impulsivity, was higher in the IA and AD groups than in the HC group (*p* < 0.001). Cognitive impulsiveness was significantly higher in the AD than in the IA and HC groups, (*p* = 0.006), although motor and nonplanning impulsiveness were significantly higher in the IA and AD groups than in the HC group (*p* < 0.001 for both).

Expression of both state and trait anger was significantly higher in the IA and AD groups than in the HC group (*p* < 0.001 for both).

The Pearson's correlation analysis revealed that the severity of IA, as measured by IAT total scores, was significantly correlated with BDI (*r* = 0.37, *p* = 0.042), agreeableness (*r* = −0.42, *p* = 0.021), BIS-11 total (*r* = 0.47, *p* = 0.009), state anger (*r* = 0.41, *p* = 0.025), and trait anger (*r* = 0.53, *p* = 0.003) scores. AD severity, as measured by AUDIT-K total scores, was significantly correlated with BDI (*r* = 0.56, *p* = 0.001), BAI (*r* = 0.51, *p* = 0.004), neuroticism (*r* = 0.38, *p* = 0.039), BIS-11 total (*r* = 0.38, *p* = 0.040), and state anger (*r* = 0.43, *p* = 0.04) scores.

## Discussion

We directly compared the personality and temperament characteristics of patients with IA with those with AD, focusing particularly on characteristics that may contribute to aggressive behaviors. Few previous studies have investigated this issue. As expected, compared with the HC group, the IA and AD groups exhibited a higher level of neuroticism and a lower level of agreeableness and extraversion, which have been associated with aggression, in terms of the Five Factor Model of personality [14]. Furthermore, the IA and AD groups exhibited higher levels of impulsivity and anger expression, characteristics linked with aggression, than did the HC group [13,14]. Although the aggressive tendency reflected by those psychological traits could not directly be interpreted as those with IA and AD usually do aggressive and risky behaviors or commit violent crimes, our findings suggest that strategies to prevent or reduce aggression in patients with AD [36] may also be important for patients with IA.

We found a striking similarity between patients with IA and those with AD in terms of emotion, temperament, and personality traits in general. Patients in both groups were more depressed and anxious than were the HCs. Moreover, patients with IA and AD had lower levels of conscientiousness, extraversion, and originality, also referred to as openness to experience, than did HCs. A previous study among college students found that students with IA showed the same pattern of deviations as did our patients with IA. Although our IA group was significantly younger than the HC group, our finding that patients with IA were more depressed than were the HCs is consistent with the results of that previous study showing that students with IA were more depressed than those without IA [15]. This suggests that the personality differences between the IA and HC groups were a function of the disorder rather than of age. We found that impulsivity was high in the IA and AD groups; however, cognitive impulsivity was higher among the patients with AD than among those with IA.

Interestingly, we found a positive linear correlation between the severity of IA and AD and a diverse range of types of psychopathology, such as depression, anxiety, impulsivity, anger, and deviant personality traits. Although a causal relationship between the severity of addictive behaviors and other deviant psychological characteristics cannot be confirmed, these findings may have important clinical implications. The management of coexisting psychopathology such as depression and anxiety is critical for the treatment of AD [37]. Similarly, clinicians should be attentive to accompanying psychopathology when treating patients with IA [38]. It is possible that the treatment of accompanying psychopathology may reduce the severity of IA, as is the case for AD.

In the present study, the three groups significantly differed in terms of age and prerequisite to perform an analysis of covariance (ANCOVA) were not met to adjust for the potentially confounding effect of age. In the Five Factor Model, younger age in early adulthood is associated with lower levels of agreeableness and conscientiousness and higher levels of extraversion and openness [39]. In this regard, lower levels of agreeableness and conscientiousness observed in patients with AD were probably not due to age because they were the oldest among the three groups. Because symptoms of depression and anxiety decline with age [40] and expression of trait and state anger are negatively correlated with age [41], it is unlikely that the more pronounced depressive and anxiety symptoms and expression of anger exhibited by our AD group were attributable to the older age of these patients. On the other hand, a previous study found that anger expression was higher among IA than among non-IA adolescents [42]. Thus the more pronounced expression of anger among patients with IA may be attributable to both the condition and younger age.

Overall, our findings indicate that IA and AD are similar in terms of personality and temperament with regard to traits that are linked to aggression and those that are not. IA has not been included as an official diagnostic classification in the DSM-IV or the tenth revision of the International Classification of Diseases (ICD-10). The DSM 5 only recently recognized IA as Internet Gaming Disorder, which is a significant component of a much broader IA category; however, the condition was not included in the formal diagnostic criteria but as a ‘condition for further study’ [43]. Although the classification of IA as an addictive disorder is controversial, our findings indicate that IA is similar to AD in terms of not only symptom patterns but also underlying personality and temperament traits, suggesting that it is plausible to group them together. However, further studies addressing underlying neurobiological mechanisms and shared treatment responses are needed to confirm a relationship between IA and AD.

The present study has several limitations. Because all participants were male, our findings may not be generalizable to females. However, this homogeneity eliminated sex as a potential confounder. We excluded subjects having IA and AD to eliminate potential ambiguity in the interpretation of our findings although coexistence of excessive Internet use and alcohol abuse is not uncommon [44]. The characteristics of patients with both addictions might differ from those of patients with either IA or AD in our study.

The present study has several strengths. Unlike most previous investigations, we studied patients who sought treatment for IA or AD. As reflected by IAT and AUDIT-K scores, both groups exhibited moderate-to-severe addictive behavior. Thus, our study has the advantage of identifying the characteristics of treatment-seeking patients with much severe psychopathology, making our findings relevant in clinical settings. The patients in previous investigations of AD were significantly older than were those in our study and, given the age difference, it is difficult to make a meaningful comparison between AD and IA. In contrast, we enrolled only young patients with AD, who were comparable to our patients with IA in terms of age, enabling a direct comparison.

## Conclusions

IA and AD are similar in terms of emotional, temperamental, and personality traits, and they share characteristics that may lead to aggression. Our findings suggest that IA and AD should be dealt with as having a close nosological relationship and that they might share common neurobiological mechanisms which may underlie their similar psychological characteristics. Further research to elucidate the nature of the relationship between IA and AD and clarify their neurobiological substrates is warranted.

## Competing interests

The authors declare that they have no competing interests.

## Authors' contributions

JYH contributed to the design of the study, analysis of data, and writing of the paper. ARG and DJ were involved in recruiting participants and collected data. SWC, JL, JYL, HYJ, and DJK contributed to theoretical interpretation and provided significant input on the manuscript. JSC contributed to the design of the project, supervision of data collection, and provision of significant input on the manuscript. All authors read and approved the final manuscript.
